# Normalization and centering of array-based heterologous genome hybridization based on divergent control probes

**DOI:** 10.1186/1471-2105-12-183

**Published:** 2011-05-21

**Authors:** Brian J Darby, Kenneth L Jones, David Wheeler, Michael A Herman

**Affiliations:** 1 Ecological Genomics Institute, Division of Biology, Kansas State University, Manhattan, KS 66506, USA; 2 Department of Biochemistry and Molecular Genetics, University of Colorado School of Medicine, Aurora, CO 80045, USA

## Abstract

**Background:**

Hybridization of heterologous (non-specific) nucleic acids onto arrays designed for model-organisms has been proposed as a viable genomic resource for estimating sequence variation and gene expression in non-model organisms. However, conventional methods of normalization that assume equivalent distributions (such as quantile normalization) are inappropriate when applied to non-specific (heterologous) hybridization. We propose an algorithm for normalizing and centering intensity data from heterologous hybridization that makes no prior assumptions of distribution, reduces the false appearance of homology, and provides a way for researchers to confirm whether heterologous hybridization is suitable.

**Results:**

Data are normalized by adjusting for Gibbs free energy binding, and centered by adjusting for the median of a common set of control probes assumed to be equivalently dissimilar for all species. This procedure was compared to existing approaches and found to be as successful as Loess normalization at detecting sequence variations (deletions) and even more successful than quantile normalization at reducing the accumulation of false positive probe matches between two related nematode species, *Caenorhabditis elegans *and *C. briggsae*. Despite the improvements, we still found that probe fluorescence intensity was too poorly correlated with sequence similarity to result in reliable detection of matching probe sequence.

**Conclusions:**

Cross-species hybridizations can be a way to adapt genome-enabled tools for closely related non-model organisms, but data must be appropriately normalized and centered in a way that accommodates hybridization of nucleic acids with diverged sequence. For short, 25-mer probes, hybridization intensity alone may be insufficiently correlated with sequence similarity to allow reliable inference of homology at the probe level.

## Background

Many organisms that are important components of most ecosystems are understudied at the genetic level because they lack useful genome-enabled resources. Hybridization of nucleic acids from non-model organisms onto DNA microarrays designed for closely related model-organisms has been used as a potential alternative to building genomic resources for each species of interest. A variety of platforms and objectives in contemporary applications of heterologous ("cross-species") hybridizations, but the recurring challenge for each platform is to measure the effect of sequence dissimilarity on hybridization between the probes being used and the nucleic acids of the species being hybridized. For example, Gilad et al. [1] tested hybridization efficiency of microarrays spotted with amplicons from four primate species (including human) and showed that increasing sequence divergence resulted in reduced hybridization efficiency. Similarly, an array of expressed sequence tags (ESTs) from African cichlid fish (*Astatotilapia burtoni*) was used to test the validity of gene expression analysis on a variety of related teleost fish [2]. The number of spots (probe features) that were able to demonstrate differential gene expression decreased with increasing phylogenetic distance. The microarrays were subsequently used to assess gene expression from swordtail (*Xiphophorus nigrensis*) [3], which was estimated to be at the far edge of what was considered phylogenetically close enough to be reliable for cross-species hybridization on the cichlid arrays. Similar arrays developed from zebrafish (*Danio*) ESTs have been used with coral reef fish (*Pomacentrus*) cDNA [4]. *In situ *synthesized oligonucleotide arrays are an alternative to spotted cDNA microarrays and commonly used when the species of interest is closely related to a model organism for which a commercially designed chip is already available. For expression studies, it is common to screen probes for sequence conservation by first hybridizing heterologous gDNA, and secondly assessing gene expression by hybridizing experimental cDNA and analyzing only the accepted probes [5]. This strategy has been applied to examine gene expression of various genera of Brassicaceae on an array containing *Arabidopsis thaliana *probes, [5-7], expression of banana genes on a rice array [8], expression of horse genes on an array containing human probes [9], and expression of goat genes using a bovine array [10].

The preparation of heterologous hybridization data for analysis is problematic because probe binding is a result of multiple factors, including binding free-energy, self-folding, dimerization, and, importantly, sequence similarity or divergence [11]. Traditional approaches to analyzing heterologous hybridization data largely follow the techniques of array-based comparative genome hybridization (aCGH ) [12-14], which is the hybridization of gDNA to con-specific arrays for the detection of chromosomal or copy-number variations. These techniques can include local regression normalization and quantile normalization. However, the conventional normalization procedures designed for aCGH have the potential to result in the false appearance of homology if the probe signals from cross-species hybridizations violates the underlying assumptions of uniform statistical distributions due to sequence divergence. Several methods have been proposed to 'screen' probes and reduce the potential for false positives [15]: 1) accept only probes of a certain hybridization fluorescence threshold or overall intensity [5,16], 2) match probes from a reference genome to that of the target genome and only analyze probes of a certain sequence similarity [17], or 3) normalize the entire dataset using a suite of known conserved genes [18,19]. However, the significant challenge with normalizing intensity data based on conserved genes is that genes evolve at different rates for different lineages. Many non-model organisms have such little genomic sequence data known that identifying sets of genes with conserved sequences amongst a group of species is unreliable, if not impossible. We propose a normalization and centering approach that relies on universally diverged (non-conserved) probes and does not make any prior assumptions about the distribution of probe signal intensities.

**Figure 1:**
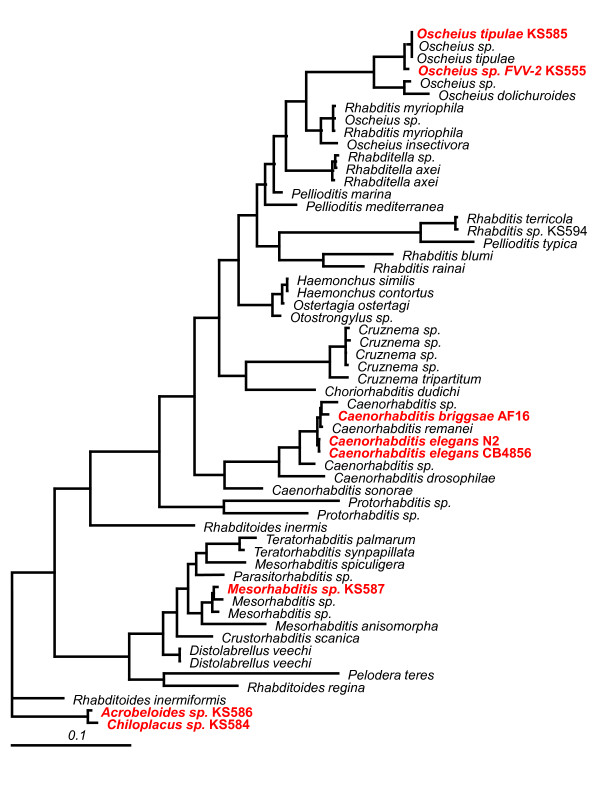
**Phylogeny of study species**. 18s ribosome RNA (small subunit) phylogeny of nematode species included in this study (in bold, red font), in comparison to 49 other species from the family Rhabditidae. *Acrobeloides *and *Chiloplacus *(from the family Cephalobidae) are used as outgroups.

Our initial objective in this study was to identify probes with conserved sequences that could be used as potential primer sites to allow amplification and subsequent cloning of orthologous genes of interest. Traditional normalization techniques led to numerous false positive matches, so we developed an algorithm for normalizing and centering intensity data of heterologous hybridization of gDNA for when the sequence divergence of species being used is unknown and a set of conserved genes is unreliable of impractical. Our approach uses built-in "control" probes from a plant (*Arabidopsis thaliana*) and a bacterium (*Bacillus subtilis*) that are available on the commercial *Caenorhabditis elegans *tiling array from Affymetrix^® ^and assumed to be equivalently diverged from all nematode species used here (from the family Rhabditidae and Cephalobidae). This procedure is most applicable to oligonucleotide microarrays with universally diverged "control" features (e.g. from a different phylum or, preferably, domain than the target species) that have been hybridized with single dye-labeled gDNA of a related, but heterologous, species. The approach is tested for sensitivity and specificity using two isolates of *C. elegans*, one isolate of *C. briggsae *(for which the full genome sequence is available), and five other nematode species with little to no known sequence data beyond small subunit ribosomal RNA gene sequences (Figure 1). These nematodes are reasonable candidates for the study of genomic responses to environmental perturbation due to their ease of study in laboratory conditions and their phylogenetic proximity to the model species *C. elegans*. Furthermore, the species we selected all co-occur on the Konza Long-Term Ecological Research station [20], so it would be likely that researchers might wish to attempt heterologous array hybridization with a similar suite of nematodes. They represent the bacterial-feeding nematodes from the tallgrass prairie biome [21] and perform key ecosystem services such as regulating bacterial turnover and nutrient cycling in soil environments [22,23]. Renn et al. [2] suggested that heterologous hybridization is most successful when the species are diverged by less than 10 MYA, but also estimated that the approach could be used on species separated by as much as ~65 MYA. Dating of the molecular divergence between these nematode species is inexact due to the lack of a robust and informative fossil record, but estimates of divergence time between *C. elegans *and *C. briggsae *has been narrowed down to 80-110 MYA following full genome sequencing [24]. Therefore, the two species from the family Cephalobidae (*Acrobeloides sp*. and *Chiloplacus sp*.) are so far diverged from *C. elegans *that they can serve as a negative control, while species within the family Rhabditidae (*Oscheius tipulae, Oscheius sp*. FVV-2, and *Mesorhabditis sp*.) serve as the species of interest to demonstrate the viability of this approach.

## Methods

### gDNA and Hybridization conditions

Strains used for hybridization included *Caenorhabditis elegans *(N2, *C. elegans *(CB4856), *C. briggsae *(AF16), and five species isolated from Konza Prairie, Riley County, Kansas (US): *Oscheius tipulae *(KS585) [Genbank:HQ130502], *Oscheius sp*. FVV-2 (KS555) [Genbank:HQ130503], *Mesorhabditis sp*. (KS587) [Genbank:HQ130505], *Acrobeloides sp*. (KS586) [Genbank:HQ130506], *Chiloplacus sp*. (KS584) [Genbank:HQ130507]. Genomic DNA was isolated from each species by phenol-chloroform extraction, labelled and hybridized onto the GeneChip^® ^*C. elegans *Tiling 1.0R Array according to manufacturer's specification using two chips per species representing biological replicates. Arrays were imaged on GeneChip^® ^Scanner 3000-7G and data extracted with GeneChip^® ^Operating Software (GCOS) and analyzed using Tilling Analysis Software (TAS). Raw and processed data has been submitted to NCBI Gene Expression Omnibus [GEO:GSE23667].

### Analysis

Raw background-subtracted probe intensities were accessed from Affymetrix^® ^Tiling Analysis Software (TAS). Subsequent analyses were performed in Statistical Analysis Software, Release 9.1.3 (SAS Institute, Cary, NC, USA). Our approach utilizes "control" probes that are standard features of the Affymetrix *C. elegans *tiling chip that correspond to sequences in the *Arabidopsis thaliana *and *Bacillus subtilis *genomes. These features are used in gene expression studies to calibrate intensity with transcript concentration and to estimate 5' and 3' end bias, but are otherwise unused in genome hybridization. Probe-specific normalization was performed by first quantifying the relationship between log_*e*_-transformed probe intensity (ln(*i*_*c,s*_)) of control probes *c *from species *s *and thermodynamic binding affinity ΔG_37 _(Gibb's free energy estimate, according to the nearest neighbour thermodynamic model of SantaLucia [25]):(1)

where α is the intercept and *e *the error term (assumed to be normally distributed). We assume that gDNA from all nematode species was equally dissimilar from these control probes, therefore subtle differences in the relationship between binding efficiency of control probes to thermodynamic binding affinity most likely represents chip to chip variation. The resulting model parameters from (1) were used to normalize probe *p *from each species *s *to its final adjusted intensity (AI):(2)

where the median intensity (median(*c,s*)) of all control probes *c *from species *s *was used as a phase shift to center all control probes around zero.

To characterize the relationship between probe intensity and the percent similarity, we make use of a dataset of candidate genes with potential ecologically relevant roles in nematode survival [26]. We selected 49 of the candidate genes of interest that had only one putative ortholog and confirmed that this suite of genes came from all six chromosomes (I: 3, II: 3, III, 6, IV: 8, V: 18, X: 11) with a group GC content (min: 40.1%, mean: 47.4%, max: 65.8%) that was representative of all probes in exon regions (43% ± 9.2 SD). We then aligned each probe from the *C. elegans *chip to its respective position in the *C. briggsae *homolog and computed the number of identical nucleotides.

## Results and Discussion

### Conventional data transformation

**Figure 2:**
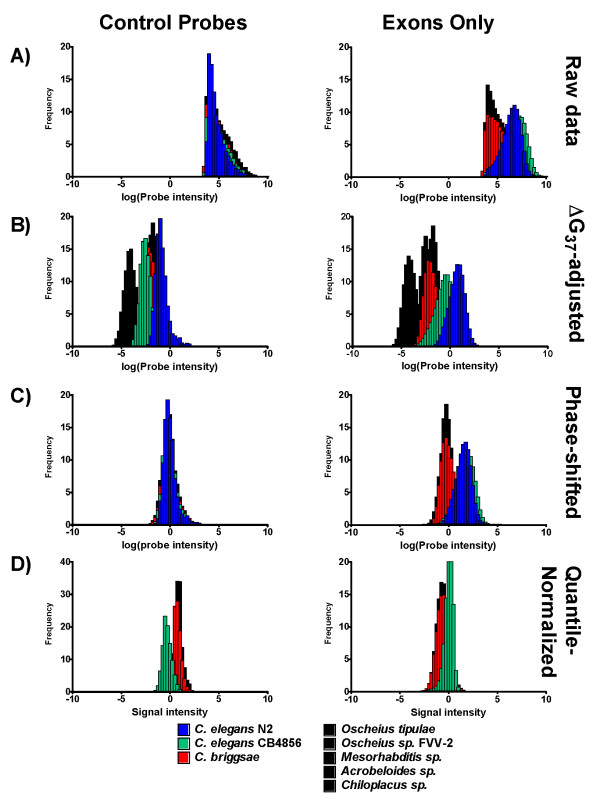
**Normalizing and centering probe intensity data**. Data transformation steps used to normalize and center data from A) initial raw probe intensity, to B) adjusted for thermodynamic binding affinity (ΔG_37 _), to C) centering of median control probes, in comparison to D) quantile normalization. Data are graphed for *Caenorhabditis *elegans N2 (blue), *C. elegans *CB4856 (green), *C. briggsae *(red), and non-Caenorhabditis species (black), but note that quantile normalized signals (D) are relative to homologous hybridization, so no data for N2 is shown. Left column: signals from control probes only (from *Arabidopsis thaliana *and *Bacillus subtilis *sequences); right column: signals from exon probes only.

We initially analyzed the hybridization data using a conventional aCGH approach that included quantile normalization [12,13], scaling on a per-array basis to a common mean (500 intensity units), and a wavelet-based smoothing of 50-bp bandwidth to 'de-noise' the data and accentuate regions of dissimilarity, or copy-number deviations, from baseline [14]. At first inspection, this conventional aCGH analysis resulted in probes of high signal intensity (comparable to that of *C. elegans *N2) for even non-*Caenorhabditis *species, especially around exonic regions (Figure 2D). However, attempts to amplify orthologous genes using PCR primers based on the exonic probes of high signal intensity (relative to *C. elegans*) were unsuccessful. It is possible that the greater probe intensity of exonic regions is not due to sequence similarity but to binding affinity, because probes in exon regions had a higher average GC-content (43% ± 9.2 SD) relative to probes in intron or intragenic regions (34% ± 10.4 SD). Thus, we hypothesized that quantile normalization of cross-species hybridization has the potential to result in the appearance of reliable data, but may in fact be a misleading representation of false positive probe matches due to artifacts during the data transformation process. Furthermore, smoothing can artificially inflate the intensity value of low-intensity probes that are adjacent to high intensity probes. Finally, the mean or median of heterologous hybridizations is naturally expected to be lower than homologous hybridizations because fewer sequences are perfect matches. Scaling all arrays to a common mean (or median) inflates non-specific hybridization intensity relative to specific hybridization intensity.

### Alternate normalization and centering

**Figure 3:**
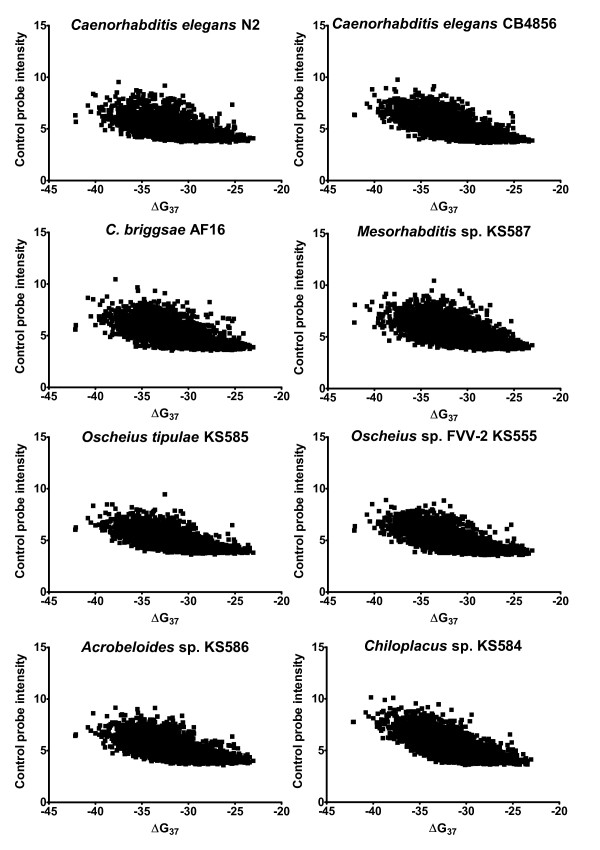
**Thermodynamics of non-specific probe binding**. The relationship between control probe intensity and thermodynamic binding affinity (ΔG_37 _) for each species tested that was used in the probe-level normalizing procedure. A) *Caenorhabditis elegans *N2, B) *C. elegans *CB4856, C) *C. briggsae*, D) *Mesorhabditis sp*., E) *Oscheius tipulae*, F) *Oscheius sp*. FVV-2, G) *Acrobeloides sp*., H) *Chiloplacus sp*.

To address the need for analyzing heterologous hybridization data with species for which the true genome-wide sequence similarity was unknown, we developed an alternative, probe-level normalization and centering. If normalization results in normally (or near normally) distributed probe intensities, the only remaining variability is likely to be predominantly random experimental error. The purpose of centering is to ensure that equivalent baseline hybridization is centered on a single common value. The raw probe intensity values (and statistical distribution) of log_*e*_-transformed control were nearly, but not exactly, similar between all chips (Figure 2A). After adjustment for the relationship between probe intensity and thermodynamic binding affinity (Figure 3), both target and control probes were uncentered, but normally distributed (Figure 2B). Finally, all probes were phase shifted to bring the median control probe intensity to zero (Figure 2C). The result was that target probes from two *Caenorhabditis elegans *isolates averaged greater than zero (indicating specific binding signal), but the target probes from non-*Caenorhabditis *species were centered near zero (indicating lack of specific binding). However, not all genes evolve at the same rates and it is possible that some genes within the genomes of the non-*Caenorhabditis *species are conserved and bind specifically to the *C. elegans *probes. If the average (normalized and centered) signal of all exon probes for a particular gene is significantly greater than zero, such a gene could reasonably be considered to be "conserved". As can be expected, the number of genes whose full set of exon probes average greater than zero decreases with increasing phylogenetic distance from *C. elegans *N2 (*C. elegans *CB4856: 17,399, *C. briggsae *AF16: 410, *Oscheius tipulae *KS585: 66, *Oschieus sp*. FVV-2 KS555: 61, *Mesorhabditis sp*. KS587: 41, *Acrobeloides sp*. KS586: 35, *Chiloplacus sp*. KS584: 30; t-test with Bonferroni correction for multiple tests). This was a first indication that sequence conservation in non-*Caenorhabditis *species may be too limited to permit detection of conserved regions.

### Test of sensitivity with con-specific hybridization

**Figure 4:**
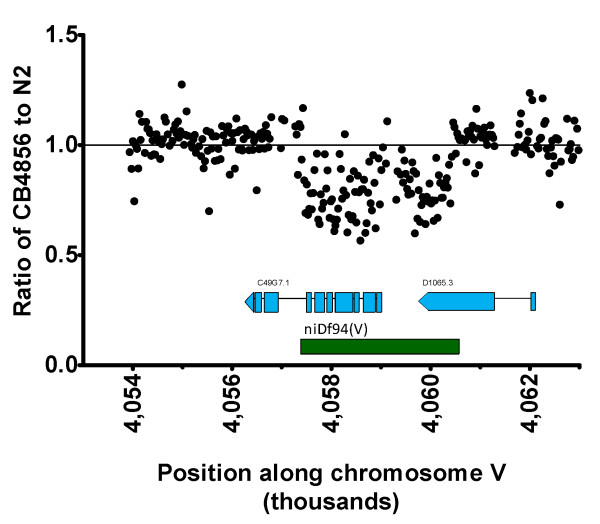
**Confirmation of hybridization sensitivity**. The ratio of *C. elegans *CB4856 to *C. elegans *N2 probe intensity values (following probe-level normalization and centering) in relation to the *niDf94(V) *deletion variant (of chromosome V), which overlaps two uncharacterized genes in the N2 genome, C49G7.1 and D1065.3a. The dip in probe intensity ratios below 1.0 is representative for the other 141 deletion variants previously identified [21].

To confirm that our normalization and centering approach was as capable of detecting conspecific variations as traditional normalization approaches, we were able to visually confirm 121 out of the 131 previously published deletions in *C. elegans *CB4856 [27]. To illustrate a representative case, Figure 4 shows a decrease in the ratio of probe values surrounding *niDf94 (V)*, a deletion of 3-kb of sequences in CB4856 (partially deleting the C49G7.1 and D1065.3 genes) that are present in N2. The few deletions we did not observe are likely due to small differences in genome coverage or different probe lengths (25 vs. 50) between the two platforms used.

### Test of specificity with cross-species hybridization

**Figure 5:**
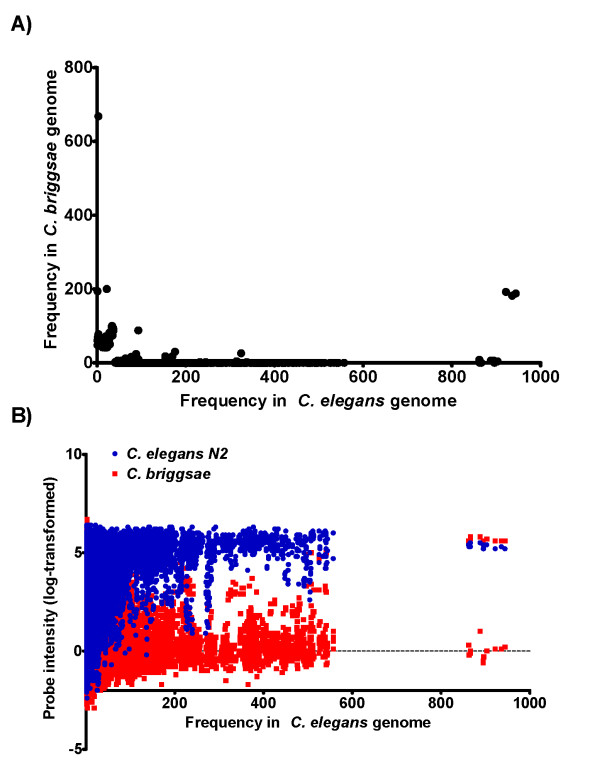
**Effect of multiple-copy probes on binding intensity**. A) Comparison of probe frequency found in *C. elegans *and *C. briggsae *genomes. B) Probe intensity of *C. elegans *(blue) and *C. briggsae *(red) gDNA related to probe frequency in *C. elegans *genome.

**Figure 6:**
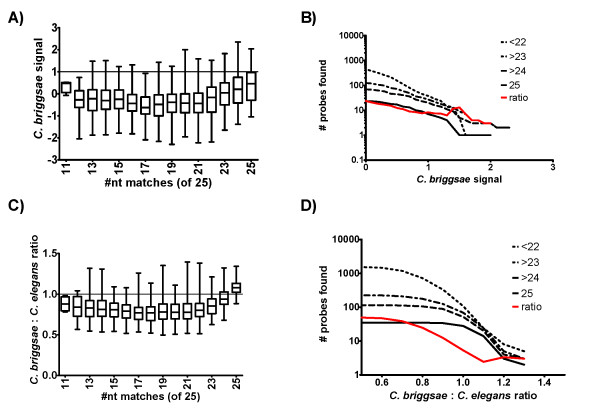
**Heterologous probe binding of *Caenorhabditis briggsae***. A) Quantile-normalized *C. briggsae *probe intensity (relative to *C. elegans *N2) as a function of probe similarity (nucleotide matches out of 25 from all probes of the 49 candidate genes with one putative ortholog). B) Accumulation of false (<22), true (25), and semi-true (>23, >24) positive probe matches at threshold ratios below 1.3 from previous panel (Figure 6A). Note that no threshold ratio is available to distinguish a greater number of semi-true positive (>23 nt) than false (<22) matches. C) and D) As in A) and B), but for alternative normalized and centered probe signal intensities proposed here. Red lines in B and D represent the ratio of mismatch probes (<25 nt) to perfect match probes (= 25 nt).

Our initial objective of designing primers from suitable probes would involve identifying probes of conserved sequence as indicated by a fluorescence signal of significant intensity relative to that of known homologous binding from *C. elegans*. In order to do this we computed the ratio of each probe's signal intensity relative to that of *C. elegans *for each heterologous test species. We assumed that any 'threshold' ratio used to define a putatively homologous probe match could result in false positive matches, so we took advantage of the fully sequenced *C. briggsae *genome to define a signal intensity ratio that would minimize the rate of incorrectly identified sequence matches. We expected two major sources of variability in the intensity of *C. briggsae *gDNA hybridized to *C. elegans*, 1) multiple occurrences of the same probe sequence within either the *C. elegans *or *C. briggsae *genome, and 2) sequence divergence between the two species. First, we computed the number of times that each 25-mer probe sequence occurred in the *C. elegans *and *C. briggsae *genomes (Figure 5A). As expected, probe intensity from *C. elegans *gDNA was positively related to frequency, while probe intensity of *C. briggsae *gDNA was generally unaffected (Figure 5B). Although many probes occur multiple times in the *C. briggsae *genome, it is a small portion of the over three million probes on the array and does not have a large effect on the overall results. Next, we determined the nucleotide similarity at each corresponding probe position between *C. elegans *and *C. briggsae *for 49 single copy genes, identified in a previous study as being potentially important for survival in different bacterial environments. Quantile normalization resulted in poor discrimination of true positive probe matches (Figure 6A) and a relative high false positive rate at all potential signal intensity ratios (Figure 6B). Our alternate normalization and centering improved the discrimination of true perfect match probes (Figure 6C), and lowered the ratio of false-matches to perfect-matches at some threshold ratios (Figure 6D). Although the values resulting from quantile normalization and from our proposed normalization and centering are comparable, they are not necessarily on equivalent scales. For example, selecting a signal intensity ratio from quantile normalization of 0.6 detects 14 true positive matches, but results in over 155 false positive mismatches. A ratio of 1.1 from our proposed alternative normalization and centering based on universally diverged control probes also detects 14 true positive matches, but results in only 34 mismatches. Despite an over 4.5-fold reduction, the ultimate false positive detection rate is still high.

## Conclusions

Cross-species hybridization has been proposed as a way to adapt genome-enabled tools developed for model organisms to closely related non-model relatives. However, we (present work) and others [18,19] have shown that the data must be appropriately normalized and centered to control for sequence divergence. Ultimately, we found that probe intensity alone was a poor predictor of sequence similarity and can result in false inferences of homology. Our findings largely support the recent results of Machado and Renn [18] who also found that the ability to detect genes decreased below 90% sequence identity between three species of *Drosophila*. The major difference in our approach is that Machado and Renn normalize based on the 100 or 1000 most conserved genes (assumed to be equivalently similar for all species of interest), while we propose normalizing and centering based on control, non-target probes (assumed to be equivalently dissimilar for all species tested). Both approaches appear to be valid for their respective purposes, but our approach might be more applicable in the absence of enough genomic sequence data to identify an *a priori *set of conserved genes. The lack of universally dissimilar probes on the spotted chip of Machado and Renn [18] prevent us from applying our technique on their data, and the lack of genomic sequence data amongst our species prevent us from applying their technique on our data. However, we can nonetheless predict that the microarrays printed with PCR products ~500 bp long [18] are likely to be more sensitive and specific to their targets than the 25-mer probes used in the Affymetrix platform presented here. Single mismatches may have a more adverse affect on the binding of short, 25-bp probes, than long, ~500 bp probes. Hybridization of gDNA onto microarrays is currently the standard technique to validate probes on gene chips for expression analysis in cross-species applications. One commonly used procedure [5] hybridizes heterologous gDNA from a non-model organism onto an existing 25-mer GeneChip^® ^designed for a model organism and masks all probe sets except those with at least one probe feature whose hybridization intensity is above a predefined threshold intensity. Our analysis suggests that, either with or without probe-level normalization and centering, a large number of non-specific control probes can still have a relatively high hybridization intensity compared to specific probes (Figure 2A). Furthermore, even if the threshold intensity were set relative to target genome hybridization, we show that a significant fraction of probe features at all threshold intensities could likely be false-positives. Thus, we fear that a cross-species hybridization algorithm to mask chips for gene expression may still permit a large number of false positive probe sets into the analysis. It is for this reason that studies utilizing cross-species hybridization for microarray gene expression profiles must be especially diligent with replication and validation. For example, Pavlidis et al. [28] found that a minimum of five biological replicates generated stable gene expression profiles. Unfortunately, recent studies using cross-species hybridization on microarrays with short probes either include no replication or insufficiently validate their microarray results with qPCR [8,9]. We suggest that cross-species microarray hybridizations introduce a degree of uncertainty beyond what is typical for con-specific hybridizations, and thus require more robust quality control measures than would be normally adopted for con-specific hybridization.

Genomic DNA controls are essential to ensure the most reliable interpretation of heterologous hybridization applications, such as gene expression profiles. Our strategy for normalization and centering of cross-species array data is meant to be used to identify reliable probe intensity values that could be utilized in downstream applications, such as finding regions of sequence similarity or for gene expression analysis. Our method is not necessarily meant to be used as a normalization procedure *per se*, although we could imagine that such an approach could be developed based on the analyses presented here. One such approach would be first to build universal control probe sets into the microarray of interest using random oligonucleotides or sequences derived from universally diverged taxa such as prokaryotes for eukaryotic arrays or *vice-versa*. Secondly, hybridize both homologous genomic DNA (from the species used to design the array) and heterologous (from the species of interest) genomic DNA onto the arrays being used (either dual-labelled mixtures onto the same chip or single-labelled pools onto separate chips) to compare probe intensity using the "control" based normalization and centering approach presented here. Finally, test the mean signal of a gene's exon probes against "zero" (with an appropriate correction for multiple comparisons). Only those genes whose complement of exon probes are statistically greater than zero can be considered "conserved" enough for use. Based upon our analyses, the number of these "conserved" genes decreases rapidly with phylogenetic distance and suggests that for distantly related taxa non-array based approaches might be more appropriate and cost effective.

## Authors' contributions

KLJ designed the project and carried out the hybridizations, BJD performed the analysis and drafted the manuscript, DW directed the analysis and assisted in interpretation of data, MAH participated in project design, and coordination of the project, and helped draft the manuscript. All authors read and approved the final manuscript.

## References

[B1] Gilad Y Rifkin SA Bertone P Gerstein M White KP Multi-species microarrays reveal the effect of sequence divergence on gene expression profiles Genome Research 2005 15 5 674 680 10.1101/gr.3335705 15867429 PMC1088295

[B2] Renn S Aubin-Horth N Hofmann H Biologically meaningful expression profiling across species using heterologous hybridization to a cDNA microarray BMC Genomics 2004 5 1 42 10.1186/1471-2164-5-42 15238158 PMC471549

[B3] Cummings ME Larkins-Ford J Reilly CRL Wong RY Ramsey M Hofmann HA Sexual and social stimuli elicit rapid and contrasting genomic responses Proceedings of the Royal Society B: Biological Sciences 2008 275 1633 393 402 10.1098/rspb.2007.1454 18055387 PMC2212751

[B4] Kassahn KS Caley MJ Ward AC Connolly AR Stone G Crozier RH Heterologous microarray experiments used to identify the early gene response to heat stress in a coral reef fish Molecular Ecology 2007 16 8 1749 1763 10.1111/j.1365-294X.2006.03178.x 17402988

[B5] Hammond J Broadley M Craigon D Higgins J Emmerson Z Townsend H White P May S Using genomic DNA-based probe-selection to improve the sensitivity of high-density oligonucleotide arrays when applied to heterologous species Plant Methods 2005 1 1 10 10.1186/1746-4811-1-10 16280083 PMC1308859

[B6] Hammond JP Bowen HC White PJ Mills V Pyke KA Baker AJM Whiting SN May ST Broadley MR A comparison of the *Thlaspi caerulescens *and *Thlaspi arvense *shoot transcriptomes New Phytologist 2006 170 2 239 260 10.1111/j.1469-8137.2006.01662.x 16608451

[B7] Morinaga SI Nagano AJ Miyazaki S Kubo M Demura T Fukuda H Sakai S Hasebe M Ecogenomics of cleistogamous and chasmogamous flowering: genome-wide gene expression patterns from cross-species microarray analysis in *Cardamine kokaiensis *(Brassicaceae) Journal of Ecology 2008 96 5 1086 1097 10.1111/j.1365-2745.2008.01392.x

[B8] Davey M Graham N Vanholme B Swennen R May S Keulemans J Heterologous oligonucleotide microarrays for transcriptomics in a non-model species; a proof-of-concept study of drought stress in Musa BMC Genomics 2009 10 1 436 10.1186/1471-2164-10-436 19758430 PMC2761422

[B9] Graham NS Clutterbuck AL James N Lea RG Mobasheri A Broadley MR May ST Equine transcriptome quantification using human GeneChip arrays can be improved using genomic DNA hybridisation and probe selection The Veterinary Journal 2010 186 3 323 327 10.1016/j.tvjl.2009.08.030 19786357

[B10] Faucon F Rebours E Bevilacqua C Helbling JC Aubert J Makhzami S Dhorne-Pollet S Robin S Martin P Terminal differentiation of goat mammary tissue during pregnancy requires the expression of genes involved in immune functions Physiol Genomics 2009 40 1 61 82 10.1152/physiolgenomics.00032.2009 19843654

[B11] Pozhitkov A Noble PA Domazet-Loso T Nolte AW Sonnenberg R Staehler P Beier M Tautz D Tests of rRNA hybridization to microarrays suggest that hybridization characteristics of oligonucleotide probes for species discrimination cannot be predicted Nucleic Acids Research 2006 34 9 10.1093/nar/gkl133 PMC1463897 16707658

[B12] Bolstad BM Irizarry RA Astrand M Speed TP A comparison of normalization methods for high density oligonucleotide array data based on variance and bias Bioinformatics 2003 19 2 185 193 10.1093/bioinformatics/19.2.185 12538238

[B13] Irizarry RA Bolstad BM Collin F Cope LM Hobbs B Speed TP Summaries of Affymetrix GeneChip probe level data Nucl Acids Res 2003 31 4 e15 10.1093/nar/gng015 12582260 PMC150247

[B14] Hsu L Self SG Grove D Randolph T Wang K Delrow JJ Loo L Porter P Denoising array-based comparative genomic hybridization data using wavelets Biostat 2005 6 2 211 226 10.1093/biostatistics/kxi004 15772101

[B15] Bar-Or C Czosnek H Koltai H Cross-species microarray hybridizations: a developing tool for studying species diversity Trends in Genetics 2007 23 4 200 207 10.1016/j.tig.2007.02.003 17313995

[B16] Degletagne C Keime C Rey B de Dinechin M Forcheron F Chuchana P Jouventin P Gautier C Duchamp C Transcriptome analysis in non-model species: a new method for the analysis of heterologous hybridization on microarrays BMC Genomics 2010 11 1 344 10.1186/1471-2164-11-344 20509979 PMC2901317

[B17] Bar-Or C Bar-Eyal M Gal T Kapulnik Y Czosnek H Koltai H Derivation of species-specific hybridization-like knowledge out of cross-species hybridization results BMC Genomics 2006 7 1 110 10.1186/1471-2164-7-110 16677401 PMC1482311

[B18] Machado H Renn S A critical assessment of cross-species detection of gene duplicates using comparative genomic hybridization BMC Genomics 2010 11 1 304 10.1186/1471-2164-11-304 20465839 PMC2876127

[B19] Renn S Machado H Jones A Soneji K Kulathinal R Hofmann H Using comparative genomic hybridization to survey genomic sequence divergence across species: a proof-of-concept from Drosophila BMC Genomics 2010 11 1 271 10.1186/1471-2164-11-271 20429934 PMC2873954

[B20] Jones KL Todd TC Wall-Beam JL Coolon JD Blair JM Herman MA Molecular Approach for Assessing Responses of Microbial-Feeding Nematodes to Burning and Chronic Nitrogen Enrichment in a Native Grassland Molecular Ecology 2006 15 9 2601 2609 10.1111/j.1365-294X.2006.02971.x 16842430

[B21] Todd TC Powers TO Mullin PG Sentinel nematodes of land-use change and restoration in tallgrass prairie Journal of Nematology 2006 38 1 20 27 19259426 PMC2586438

[B22] Freckman DW Bacterivorous Nematodes and Organic-Matter Decomposition Agriculture Ecosystems & Environment 1988 24 1-3 195 217 10.1016/0167-8809(88)90066-721598776

[B23] Ferris H Bongers T Nematode Indicators of Organic Enrichment Journal of Nematology 2006 38 1 3 12 19259424 PMC2586436

[B24] Stein LD Bao Z Blasiar D Blumenthal T Brent MR Chen N Chinwalla A Clarke L Clee C Coghlan A The Genome Sequence of *Caenorhabditis briggsae*: A Platform for Comparative Genomics PLoS Biol 2003 1 2 e45 14624247 10.1371/journal.pbio.0000045 PMC261899

[B25] SantaLucia J A unified view of polymer, dumbbell, and oligonucleotide DNA nearest-neighbor thermodynamics Proceedings of the National Academy of Sciences of the United States of America 1998 95 4 1460 1465 10.1073/pnas.95.4.1460 9465037 PMC19045

[B26] Coolon JD Jones KL Todd TC Carr BC Herman MA *Caenorhabditis elegans *Genomic Response to Soil Bacteria Predicts Environment-Specific Genetic Effects on Life History Traits PLoS Genetics 2009 5 6 e1000503 10.1371/journal.pgen.1000503 19503598 PMC2684633

[B27] Maydan JS Flibotte S Edgley ML Lau J Selzer RR Richmond TA Pofahl NJ Thomas JH Moerman DG Efficient high-resolution deletion discovery in *Caenorhabditis elegans *by array Comparative Genomic Hybridization Genome Research 2007 17 3 337 347 10.1101/gr.5690307 17267812 PMC1800925

[B28] Pavlidis P Li Q Noble WS The effect of replication on gene expression microarray experiments Bioinformatics 2003 19 13 1620 1627 10.1093/bioinformatics/btg227 12967957

